# Performance of PREMM5, clinical criteria, and immunohistochemistry for MMR proteins in genetic risk assessment of Mexican patients with colorectal cancer

**DOI:** 10.1371/journal.pone.0354795

**Published:** 2026-08-04

**Authors:** José Luis Rodríguez-Olivares, Dione Aguilar-y-Méndez, Tamara N. Kimball, Pamela Rivero-García, Javier Rios-Valencia, Sandra Santuario-Facio, Augusto Rojas-Martinez, Rocío Ortiz-López, Angélica Leticia Barraza-Arellano, Alejandro Aranda-Gutierrez, Jazmín Arteaga-Vazquez, Héctor De-La-Mora-Molina, Josef Herzog, Joanne M. Jeter, Jeffrey N. Weitzel, Yanin Chávarri-Guerra

**Affiliations:** 1 Department of Hematology and Oncology, Instituto Nacional de Ciencias Médicas y Nutrición Salvador Zubirán, Mexico City, Mexico; 2 Hospital Zambrano Hellion Tec Salud, San Pedro Garza García, Mexico; 3 Tecnológico de Monterrey. Escuela de Medicina y Ciencias de la Salud. Monterrey, Mexico; 4 Center for Genomic Medicine, Massachusetts General Hospital, Boston, Massachusetts, United States of America; 5 Department of Medical Genetics, Instituto Nacional De Ciencias Médicas y Nutrición Salvador Zubirán, Mexico City, Mexico; 6 Department of Pathology, Instituto Nacional de Ciencias Médicas y Nutrición Salvador Zubirán, Mexico City, Mexico; 7 Division of Clinical Cancer Genomics, Department of Population Sciences, City of Hope Cancer Center, Duarte, California, United States of America; 8 Department of Medical Oncology, City of Hope Cancer Center, Duarte, California, United States of America; 9 Division of Precision Prevention, University of Kansas Comprehensive Cancer Center, Kansas City, United States of America; PLOS ONE, UNITED KINGDOM OF GREAT BRITAIN AND NORTHERN IRELAND

## Abstract

**Background:**

All individuals with colorectal cancer (CRC) should undergo genetic cancer risk assessment given its implications for personalized treatment, surveillance, risk-reduction strategies, and cascade testing. Universal screening using immunohistochemistry (IHC) for mismatch repair (MMR) proteins in tumor tissue, when combined with clinical criteria, is essential for identifying individuals at higher risk for carrying germline pathogenic variants (PVs) in resource limited countries.

**Patients and Methods:**

The spectrum of PVs in cancer susceptibility genes was characterized using NGS multigene panel assays among selected patients with CRC at two centers in Mexico. Germline genetic testing was used as the reference standard to evaluate the diagnostic accuracy of the referral criteria.

**Results:**

From September 2018 to October 2024, 208 patients were enrolled. The median age at diagnosis was 45.0 years; 52.4% were women, 48.6% had deficient-MMR CRC, and 38.0% reported family history of CRC. Germline PVs were identified in 32.2% (n = 67); of these, 77.6% (n = 52) had Lynch syndrome (30 *MLH1*, 14 *MSH2*, 6 *MSH6*, and 2 *PMS2*), while 22.4% (n = 15) harbored PVs in other genes (4 *ATM*, 3 *BRCA1*, 3 *CHEK2*, 2 *TP53*, 1 *BRCA2*, 1 *BRIP1*, and 1 *PALB2*). Additionally, one individual harbored a monoallelic PV in *MUTYH*. The Amsterdam II criteria demonstrated the highest specificity (Sensitivity 56.0%, Specificity 91.9%), while the revised Bethesda criteria (Sensitivity 98.1%, Specificity 19.9%) and IHC for MMR proteins (Sensitivity 91.2%, Specificity 68.7%) exhibited high sensitivity. The area under the ROC curve for PREMM5 was 0.822 (95% CI 0.746–0.898).

**Conclusion:**

PREMM5, Revised Bethesda criteria and IHC for MMR proteins effectively prioritized patients eligible for germline genetic testing in resource-limited settings. The performance of PREMM5 in this Mexican cohort was comparable to findings reported in validation studies within other populations. Considering the spectrum of PVs identified, employing a multigene panel test is recommended for Mexican patients with CRC.

## Introduction

Genetic Cancer Risk Assessment (GCRA) has become an integral component of colorectal cancer (CRC) care. GCRA refers to a structured clinical process that includes: systematic collection of personal and family cancer history, risk stratification based on established clinical criteria and prediction models (e.g., Amsterdam and Bethesda criteria, PREMM5, and tumor-based testing such as immunohistochemistry), and determination of eligibility for germline testing. When indicated, this process is followed by germline testing to identify pathogenic or likely pathogenic variants (PVs) in cancer susceptibility genes. Through this comprehensive approach, GCRA informs risk-adapted surveillance, preventive interventions, therapeutic decision-making, and cascade testing for at-risk relatives [1].

CRC ranks as the third most prevalent cancer worldwide [2]. Although most early-onset cases are sporadic [3,4], and the disease is multifactorial [5], the rising incidence of CRC in younger populations underscores the need to refine strategies for identifying individuals at increased hereditary risk and to expand access to genetic services [6].

The clinical relevance of hereditary CRC syndromes, particularly Lynch syndrome (LS), has driven the development of risk stratification tools to guide germline testing. Early frameworks, including the Amsterdam I criteria, prioritized family history of CRC but failed to capture extracolonic malignancies [7]. Subsequent refinements, such as the Amsterdam II and revised Bethesda criteria, incorporated a broader tumor spectrum and molecular features, including high microsatellite instability (MSI-H), to improve case detection [8–10].

LS arises from germline PVs in DNA mismatch repair (MMR) genes (*MLH1*, *PMS2*, *MSH2*, and *MSH6*) or *EPCAM*, resulting in loss of MMR protein function and a hypermutator phenotype. This biological basis underpins the use of immunohistochemistry (IHC) as a tumor-based screening approach [11,12].

The PREdiction Model for gene Mutations 5 (PREMM5) integrates personal and family history to estimate the probability of carrying a germline PV in LS-associated genes. A threshold of ≥2.5% has demonstrated high sensitivity; however, the limited representation of Hispanic/Latino populations in validation cohorts highlights a critical gap in the generalizability of these tools [13].

Given that genetic ancestry and environmental exposures may influence CRC risk, the performance of established prediction models may not be uniform across populations. In this context, we aimed to evaluate the performance of clinical criteria, PREMM5, and IHC for identifying carriers of germline PVs in a selected cohort of patients with CRC at two centers in Mexico.

## Methods

### Study participants and genetic cancer risk assessment

From September 1, 2018, to October 28, 2024, Mexican patients were enrolled at the Instituto Nacional de Ciencias Médicas y Nutrición Salvador Zubirán (INCMNSZ) in Mexico City, as part of the CCGCRN (Clinical Cancer Genomics Community Research Network) [14–16], and at Hospital Zambrano Hellion in Monterrey, as part of the CHIBCHA project (Common Hereditary Bowel Cancers in Hispania and the Americas). The study protocol was approved by the institutional review boards of both institutions [IRB# HEM-1900 and IRB# CMN2012–001/R-2012-785-032, respectively].

The inclusion criteria were ≥18 years of age and a histopathological diagnosis of CRC, and at least one of the following criteria: age at CRC diagnosis <50 y; second primary CRC (synchronous or metachronous) regardless of age at onset; history of CRC or other LS-associated neoplasms in ≥1 first- or second-degree relatives before age 50, or ≥2 relatives regardless of age; a calculated probability score of ≥2.5% as determined by the PREMM5 predictive model; or loss of one or more DNA MMR proteins as determined by IHC on tumor tissue. Patients who met the Adenomatous Polyposis Testing Criteria (S1 Table) were excluded. These criteria functioned as a pre-screening strategy applied to the overall CRC population at the participating institutions, whereby only patients meeting at least one predefined risk factor were eligible for enrollment. Eligible individuals provided written informed consent before undergoing study procedures, including construction of a multigenerational family pedigree focused on cancer history, authorization for clinical data collection, and collection of a blood sample for germline genetic testing. Clinical data — including age at first CRC diagnosis, primary tumor site [right, left (proximal or distal to the splenic flexure, respectively [17]) or rectum], American Joint Committee on Cancer (AJCC) TNM Staging (8th ed., 2017), IHC results for MMR proteins (MLH1, PMS2, MSH2, and MSH6), and personal history of other neoplasms — were obtained from medical records.

### Next-generation sequencing and variant characterization

Multigene panel testing included the following cancer susceptibility genes: *BRCA1*, *BRCA2*, *ATM*, *CHEK2*, *PALB2*, *CDKN2A*, *RAD50*, *RAD51C*, *RAD51D*, *BRIP1*, *NBN*, *PTEN*, *CDH1*, *TP53*, *NF1*, *APC*, *STK11*, *MUTYH*, *MLH1*, *PMS2*, *MSH2*, *MSH6* and *EPCAM*. A peripheral blood sample was sequenced using an Illumina HiSEQ Genetic Analyzer. Full sequencing libraries were prepared using the KAPA Hyper library preparation kits and hybridized bar-coded samples to a custom Agilent SureSelect (Santa Clara, CA) targeted gene capture kit. The bait design included full exon coverage for multigene capture, encompassing both 5’ and 3’ untranslated regions, with sequencing of 10 base pairs into all introns for an average coverage of 300-500X. Exons 11–15 in *PMS2,* located in the pseudogene, and the *PTEN* promoter region were not fully covered. *BRCA1, MLH1, MSH2* and *MSH6* were also analyzed for copy-number variants using multiplex ligation-dependent probe amplification (MLPA; Holland). Sanger re-sequencing was used to confirm PVs. Variants of uncertain significant results or apparent polymorphisms were not reported. For this analysis, only pathogenic and likely pathogenic variants were reported according to the American College of Medical Genetics and Genomics, Association for Molecular Pathology consensus criteria, and International Agency for Research on Cancer guidelines [18]. Monoallelic PVs in *MUTYH* were deemed non-actionable and were classified as uninformative findings for the purpose of this analysis.

### Statistical analysis

Statistical analysis was performed using STATA version 17.0 software (StataCorp) and R-4.3.2 (R Core Team 2023) software. The probands were grouped based on carrier status. Descriptive statistics, including frequency and proportions for categorical variables and median and range for quantitative variables, were calculated. Mann-Whitney U and Fisher exact tests were used to examine differences in continuous and categorical variables between groups (non-carriers vs carriers of PVs in LS-associated genes), as appropriate. Statistical significance was defined as a two-sided p-value of <0.05. The diagnostic accuracy of the predictive tools was assessed using genetic testing results as the reference standard. The area under the ROC curve for the PREMM5 model was calculated to differentiate individuals with and without LS.

## Results

### Cohort characteristics

**Fig 1:**
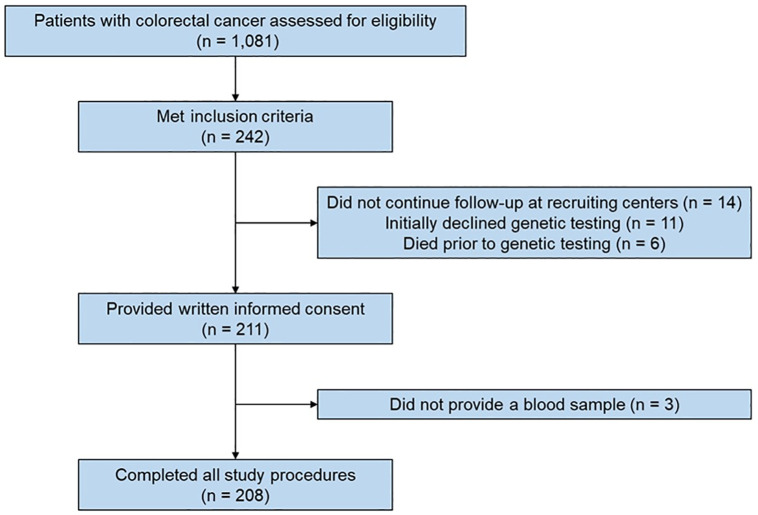
Flowchart of patient selection.

A total of 1,081 patients were assessed for eligibility, of whom 242 met the selection criteria. Among these, 211 provided written informed consent, and 208 completed all study procedures (Fig 1). Among the 208 patients included in this analysis, 127 (61.0%) were included at INCMNSZ and 81 (39.0%) at Hospital Zambrano Hellion. The distribution of clinicopathological characteristics is presented in Table 1. The median age at first CRC diagnosis for the entire cohort was 45.0 years (18.0–82.0), with 52.4% of the participants being women. The proportion of probands by stage of CRC were 13.0% for stage I, 25.5% for stage II, 21.6% for stage III, and 18.8% for stage IV. 21.1% had an unknown stage or were undergoing staging at the time of enrollment.

**Table 1:** Clinicopathological differences between non-carriers and carriers of germline PVs in Lynch syndrome-associated genes. The characteristics of carriers of PVs in other cancer susceptibility genes (*TP53, ATM, CHEK2, BRCA1, BRCA2, PALB2,* and *BRIP1*) are also described. For this analysis, monoallelic PVs in *MUTYH* were considered a non-actionable result.

Characteristic	Study population N = 208 (%)	Non carriers n=141 (%)	LS genes n=52 (%)	P^*^	Other genes n=15 (%)
Median age at first CRC diagnosis (Range)	45.0 (18.0-82.0)	47.0 (18.0-82.0)	41.0 (18.0-74.0)	**0.011**	43.0 (26.0-68.0)
Sex					
Female	109 (52.4)	78 (55.3)	22 (42.3)	0.143	9 (60.0)
Male	99 (47.6)	63 (44.7)	30 (57.7)		6 (40.0)
CRC site, No. (%)					
Right	92 (44.2)	55 (39.0)	33 (63.5)	**0.005**	4 (26.7)
Left	80 (38.5)	57 (40.4)	16 (30.8)		7 (46.7)
Rectum	29 (13.9)	23 (16.3)	3 (5.7)		3 (20.0)
Unavailable	7 (3.4)	6 (4.3)	0		1 (6.7)
Stage, No. (%)					
I	27 (13.0)	18 (12.8)	8 (15.4)	0.315	1 (6.7)
II	53 (25.5)	32 (22.7)	15 (28.8)		6 (40.0)
III	45 (21.6)	28 (19.8)	14 (26.9)		3 (20.0)
IV	39 (18.8)	29 (20.6)	7 (13.5)		3 (20.0)
Unavailable	44 (21.1)	34 (24.1)	8 (15.4)		2 (13.3)
IHC analysis for MMR protein expression					
Deficient (dMMR)^a^	52/107 (48.6)	21/67 (31.3)	31/34 (91.2)	**<0.001**	0/6
Proficient (pMMR)^a^	55/107 (51.4)	46/67 (68.7)	3/34 (8.8)		6/6 (100.0)
Unavailable	101	74	18		9
Other self-reported malignancy, No. (%)					
Any	57 (27.4)	29 (20.6)	22 (42.3)	**0.003**	6 (40.0)
Synchronous (colon)	2 (1.0)	1 (0.7)	1 (1.9)	0.467	0
Metachronous (colon)	10 (4.8)	1 (0.7)	7 (13.5)	**0.005**	2 (13.3)
Prostate^b^	8/99 (8.1)	8/63 (12.7)	0	NA	0
Endometrial^c^	8/109 (7.3)	4/78 (5.1)	3/22 (13.6)	0.177	1/9 (11.1)
Ovarian^c^	3/109 (2.8)	1/78 (1.3)	1/22 (4.5)	0.393	1/9 (11.1)
Breast	9 (4.3)	4 (2.8)	3 (5.8)	0.389	2 (13.3)
Thyroid	6 (2.9)	5 (3.5)	1 (1.9)	1.000	0
Gastric	5 (2.4)	1 (0.7)	4 (7.7)	**0.019**	0
Sebaceous glands	3 (1.4)	0	3 (5.8)	NA	0
Kidney	3 (1.4)	2 (1.4)	1 (1.9)	1.000	0
Pancreatic	2 (1.0)	2 (1.4)	0	NA	0
Others	7 (3.4)	6 (4.3)	1 (1.9)	0.676	0

* P value comparing non-carriers versus carriers of germline PVs in LS-associated genes.

a. The proportion of DNA mismatch repair protein expression status was estimated only in the group of patients with available IHC results.

b. The proportion of second primary prostate cancer was estimated only in men.

c. The proportion of second primary endometrial and ovarian malignancies was estimated only in women.

Abbreviations: LS = Lynch Syndrome, CRC = Colorectal Cancer, IHC = Immunohistochemistry, MMR = Mismatch Repair, NA = not applicable due to complete separation of estimates

LS genes include: *MLH1, PMS2, MSH2*, and *MSH6*.

### Variant detection and clinicopathological characteristics by carrier status

**Fig 2:**
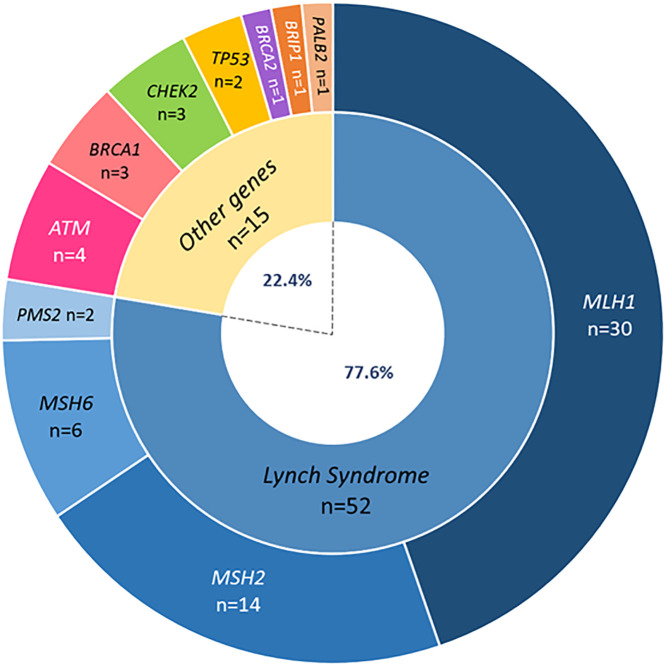
Distribution of germline pathogenic and likely pathogenic variants among Mexican patients with CRC referred for GCRA.

Germline PVs were identified in 32.2% (67/208) of the probands. Among these, 77.6% (n = 52/67) were carriers of PVs in LS-related genes (30 in *MLH1*, 14 in *MSH2*, 6 in *MSH6*, and 2 in *PMS2*). Of note, no PVs were found in *EPCAM*. 22.4% (n = 15/67) of carriers harbored PVs in other cancer susceptibility genes (4 in *ATM*, 3 in *BRCA1*, 3 in *CHEK2*, 2 in *TP53*, 1 in *BRCA2*, 1 in *BRIP1*, and 1 in *PALB2*). Additionally, one individual harbored a monoallelic PV in *MUTYH*, and was classified within the subgroup of patients with negative results. Fig 2.

Comparing non-carriers, patients with LS presented with their first CRC at an earlier age (47.0 vs. 41.0 years, p = 0.011) and had a higher proportion of primary tumors in the right colon (39.0% vs. 63.5%, p = 0.005).

IHC results for MMR proteins in tumor tissue were available for 107 individuals. Compared to individuals without germline PVs, those with LS exhibited a significantly higher frequency of MMR-deficient tumors (31.3% vs. 91.2%, p < 0.001). Individuals with LS also showed an increased occurrence of second primary malignancies (20.6% vs. 42.3%, p = 0.003), primarily due to a higher incidence of metachronous CRC (0.7% vs. 13.5%, p = 0.005) and gastric cancer (0.7% vs. 7.7%, p = 0.019). In patients with LS and known MMR status, IHC was concordant with germline test results in 91.2% of cases (31/34). The three discordant cases included one carrier each of PVs in *MLH1*, *MSH2* and *MSH6*, all of whom exhibited MMR-proficient tumors.

In the subgroup of patients with PVs in other cancer susceptibility genes, 86.7% (n = 13/15) harbored PVs in genes involved in the homologous recombination repair pathway. Among these individuals, the median age at diagnosis of the first CRC was 43.0 years; 60.0% were female, and 66.7% had tumors located in the left colon or rectum. Additionally, 20.0% presented with metastatic disease at diagnosis, and all patients with available IHC results exhibited MMR-proficient tumors.

### Clinical genetic referral criteria and prediction models performance

Family history data revealed that 38.0%, 11.5%, and 9.6% of the entire cohort reported familial occurrences of colorectal, gastric, and endometrial cancers, respectively. Compared to non-carriers, individuals with LS were significantly more likely to report a family history of colorectal (24.8% vs. 75.0%, p < 0.001), endometrial (5.0% vs. 23.0%, p = 0.005), gastric (4.3% vs. 32.7%, p < 0.001), and pancreatic cancer (3.5% vs. 13.5%, p = 0.018). Among individuals carrying PVs in other genes, 33.3% reported a family history of CRC. Table 2.

**Table 2:** Differences in self-reported family cancer history limited to first-degree and second-degree relatives on the affected side between non-carriers and carriers of PVs in LS genes.

Cancer, No. (%)	Study population N = 208 (%)	Non carriers n=141 (%)	LS genes n=52 (%)	P^*^	Other genes n=15 (%)
Colorectal	79 (38.0)	35 (24.8)	39 (75.0)	**<0.001**	5 (33.3)
Endometrial	20 (9.6)	7 (5.0)	12 (23.0)	**0.005**	1 (6.7)
Gastric	24 (11.5)	6 (4.3)	17 (32.7)	**<0.001**	1 (6.7)
Small intestine	1 (0.5)	1 (0.7)	0	NA	0
Pancreatic	13 (6.3)	5 (3.5)	7 (13.5)	**0.018**	1 (6.7)
Biliary tract	2 (1.0)	1 (0.7)	0	NA	1 (6.7)
Urinary tract	3 (1.4)	3 (2.1)	0	NA	0
Ovarian	11 (5.3)	5 (3.5)	4 (7.7)	0.254	2 (13.3)
Sebaceous glands	1 (0.5)	0	1 (1.9)	NA	0
Central nervous system	4 (1.9)	1 (0.7)	3 (5.8)	0.060	0

* P value comparing non-carriers versus carriers of germline PVs in LS-associated genes.

Compared to non-carriers, a greater proportion of individuals with LS met the Amsterdam II criteria (7.8% vs. 53.8%). Similarly, a higher percentage of LS carriers fulfilled the revised Bethesda criteria (80.1% vs. 98.0%). Additionally, 88.5% of patients with LS had a PREMM5 score ≥2.5%, as detailed in S2 Table.

The Amsterdam II criteria demonstrated good accuracy (Sensitivity 56.0%, 95% CI 41.3–70.0; Specificity 91.9%, 95% CI 85.9–95.9), while the Revised Bethesda criteria (Sensitivity 98.1%, 95% CI 89.7–99.9), PREMM5 score ≥2.5% (Sensitivity 92.0%, 95% CI 80.7–97.8), and IHC for MMR proteins (Sensitivity 91.2%, 95% CI 76.3–98.1) exhibited high sensitivity. Table 3.

**Table 3:** Diagnostic test indicators for Lynch syndrome.

Test	Sensitivity (95% CI)	Specificity (95% CI)	PPV (95% CI)	NPV (95% CI)
Amsterdam II	56.0% (41.3–70.0)	91.9% (85.9–95.9)	0.72 (0.58–0.83)	0.85 (0.80–0.88)
Revised Bethesda	98.1% (89.7–99.9)	19.9% (13.6–27.4)	0.31 (0.29–0.33)	0.96 (0.79–0.99)
IHC analysis for MMR protein expression	91.2% (76.3–98.1)	68.7% (56.2–79.4)	0.60 (0.50–0.68)	0.94 (0.84–0.97)
PREMM5 ≥ 2.5%	92.0% (80.7–97.8)	20.7% (14.3–28.6)	0.31 (0.27–0.33)	0.87 (0.72–0.95)

PPV = Positive Predictive Value, NPV = Negative Predictive Value, CI = confidence interval.

**Fig 3:**
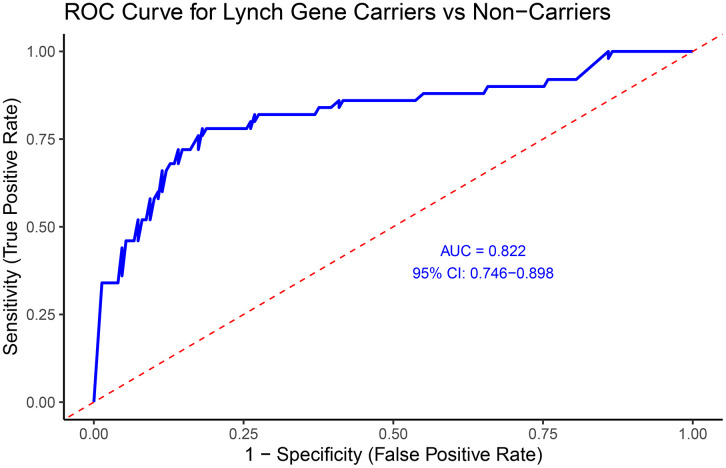
Receiver operating characteristic (ROC) curve and area under the curve (AUC) for the PREMM5 predictive model.

The area under the ROC curve for PREMM5 was 0.822 (95% CI 0.746–0.898) with a median score of 28.5% (95% CI 23.4–33.5) in patients with LS and of 5.9% (95% CI 4.1–7.6) in non-carriers. Fig 3.

## Discussion

The yield of germline PVs in our cohort of individuals with CRC who met clinical criteria for GCRA was 32.2%. In cohorts of patients with CRC selected for age younger than 50 years (with a 10% incidence of MMR-deficient tumors) a prevalence of PVs ranging from 16% to 25% has been reported [3,4]. In contrast, studies using universal multigene panel testing strategies (regardless of age at onset or family history) have reported germline PV frequencies ranging from 5% to 15% in CRC cohorts [19–21]. The high frequency of germline PVs in our cohort can likely be attributed to the highly selected nature of the population, characterized by a median age of 45.0 years at first CRC diagnosis, 48.5% presenting with MMR-deficient tumors among those with available IHC results, and 38.0% reporting a family history of CRC. Our cohort represents a referral-enriched population, which likely contributed to the high prevalence of germline PVs and may overestimate the diagnostic performance of the evaluated tools. Therefore, these findings should not be extrapolated to unselected CRC populations.

In our study, 77.6% of selected hereditary CRC cases were attributed to Lynch syndrome, predominantly involving PVs in *MLH1* (57.7% of LS cases). This aligns with a retrospective analysis of Mexicans referred to GCRA, where LS was the most confirmed hereditary syndrome in the subgroup of patients with CRC, mostly linked to *MLH1* PVs [22]. Our findings also reinforce prior evidence from Mexican LS families, in which *MLH1* was the most frequently altered gene across a broad tumor spectrum. In these families, CRC was the most prevalent cancer, highlighting its role as a frequent trigger for GCRA in this population [23].

Resources for germline testing in Mexico are limited; therefore, determining the most effective screening strategies is essential. A pivotal prospective study of 1,066 unselected individuals with newly diagnosed CRC reported that 12.7% had high microsatellite instability, and IHC demonstrated a sensitivity of 93.2% (95% CI: 88.9–97.4) for detecting tumors with this phenotype [24]. In patients with MLH1/PMS2 deficiency identified through tumor tissue IHC, other etiologies beyond LS may be responsible, such as somatic variants [25], *MLH1* promoter methylation [26], and *MLH1* constitutional methylation [27]. Among our patients with LS and available IHC results, 91.2% had MMR-deficient CRC, consistent with prior reports indicating that approximately 90.0% of individuals with LS had MMR-deficient tumors [28,29]. In contrast, 8.8% of individuals with LS-associated CRCs in our cohort were MMR-proficient, consistent with reports from other studies showing rates as high as 10.0%. These findings suggest that such patients may remain at risk of CRC through alternative pathways [29].

Universal screening through IHC for MMR proteins in tumor tissue not only identifies individuals at risk for hereditary cancer but also has significant clinical implications, such as assessing recurrence risk to guide adjuvant chemotherapy decisions in early-stage CRC [30], and selecting eligible patients for immunotherapy in neoadjuvant [31,32], adjuvant [33], and metastatic settings [34,35]. Looking ahead, the use of immunotherapy in MMR-deficient CRC is expected to play a key role in the development of organ-preserving strategies, with the goal of optimizing patient outcomes while minimizing treatment-related morbidity [36]. A high proportion of our enrolled patients had deficient-MMR tumors, suggesting a referral bias, with patients displaying this biomarker being more frequently referred to GCRA.

Among the predictors evaluated, the Amsterdam II criteria demonstrated the lowest sensitivity, likely due to their stringent requirements, which necessitate simultaneous fulfillment of multiple criteria to identify an individual as an at-risk proband. Consequently, this may result in the non-identification of individuals with limited family history [37], young carriers who have not yet manifested symptoms, carriers with lower penetrance PVs [38], and in rare instances, de novo germline PVs [39].

Previous reports indicate that approximately 72.0% of patients with LS identified through a CRC diagnosis met the revised Bethesda criteria [28]. In our cohort, 86.0% of all referred patients and 98.0% of those diagnosed with LS met these criteria, highlighting their sensitivity and suggesting they can be effectively used as referral criteria for germline genetic testing by healthcare professionals managing individuals diagnosed with CRC.

In our study, PREMM5 effectively distinguished carriers of PVs in LS-genes, achieving an area under the ROC curve (AUC) of 0.822 (95% CI 0.746–0.898) in a Mexican population. This performance is comparable to the AUC of 0.83 (95% CI: 0.75–0.92) reported by Kastrinos et al. in a cohort composed predominantly of non-Hispanic White individuals [13].

Screening strategies combining IHC for MMR proteins with germline genetic testing have been recognized as cost-effective, particularly as the number of family members undergoing genetic testing increases [40]. Enhancing access to cascade testing for targeted variant identification poses a critical challenge in Mexico, especially as the diagnosis of hereditary CRC in probands continues to rise. It may be that population genomic screening of high-evidence genes linked to hereditary conditions, including LS, could prove cost-effective with adequate access to risk-reduction measures [41]. This highlights the critical need for establishing specialized clinics to manage patients at high risk for CRC and other neoplasms.

In our cohort, 22.4% of the identified PVs were located in non-LS genes, predominantly involving genes related to homologous recombination pathway. Analyzing this subgroup was challenging due to the heterogeneous CRC risk associated with these genes. Among those identified with PVs in non-Lynch syndrome genes, only *TP53* has an established association with CRC risk. In the LIFT UP study, it was reported that *TP53* PV carriers had a 7.2% cumulative risk of CRC by age 80, with an elevated relative risk peaking at age 20 and declining after age 70 [42]. These findings support early screening colonoscopy in young adults with Li-Fraumeni syndrome. In contrast, the NCCN recently revised its guidelines [1], no longer recommending high-risk colon cancer screening for *CHEK2* PV carriers. The presence of germline PVs in non-LS genes underscores the increasing complexity of GCRA in the era of multigene panel testing. This expanded spectrum challenges traditional risk prediction tools and highlights the need to validate broader models—such as PREMMplus [43]—in our population. Such tools may improve the identification of individuals carrying PVs in a wider array of cancer susceptibility genes beyond those classically linked to CRC.

### Limitations

This study has several limitations. The cohort represents a referral-enriched population, as patients were pre-selected based on clinical criteria, abnormal IHC, or elevated risk scores. This selection approach likely increased the prevalence of germline PVs and may have overestimated the diagnostic performance of the evaluated tools, limiting generalizability to unselected CRC populations. IHC data were unavailable for nearly half of participants, which may have introduced verification bias and affected performance estimates; nevertheless, exploratory analyses showed no significant differences between patients with known and unknown MMR status (S3 Table).

A limitation of our sequencing approach is the incomplete coverage of *PMS2* exons 11–15 and the *PTEN* promoter region. *PMS2* contains regions of high homology with its pseudogene (*PMS2CL*), particularly across exons 11–15, which complicates sequencing and may lead to underdetection of PVs [44]. In addition, alterations in *PTEN* may occur through promoter methylation rather than coding sequence variants [45], which would not be captured by our approach.

The findings are derived from a cohort of Mexican patients with access to specialized centers, and therefore may not be directly generalizable to other populations with different genetic backgrounds, healthcare systems, or levels of access to genetic services.

## Conclusion

In our cohort, composed entirely of patients self-identified as having Mexican-Mestizo ancestry, we validated the accuracy of the PREMM5 model, supporting its use as a tool to guide patient selection for germline genetic testing. While no predictive model is infallible, our findings suggest that in Mexican patients with CRC, decisions regarding genetic testing should be informed—but not solely determined—by risk models.

We recommend a comprehensive clinical evaluation incorporating detailed family cancer history, physical examination, and relevant endoscopic and histological findings. In this context, use of the PREMM5 score and the revised Bethesda criteria—both demonstrating high sensitivity—may help reduce missed high-risk individuals who could benefit from germline testing. Although genetic testing for all patients with CRC should ideally become standard practice in Mexico, optimizing referral criteria remains essential to improve risk stratification in settings with limited access.

Finally, genetic testing should not be restricted to patients with MMR-deficient tumors. Although Lynch syndrome is the most common hereditary cancer syndrome in Mexican patients with CRC, our findings demonstrate a broader spectrum of germline pathogenic variants.

## Supporting information

S1 Table Adenomatous Polyposis Testing Criteria (NCCN Guidelines®). For this study, patients who fulfilled any of these criteria were excluded.
(DOCX)

S2 Table Family history-based criteria and clinical prediction models performance. (DOCX)

S3 Table Differences between patients with known versus unknown MMR status as determined by immunohistochemistry. (XLSX)
