# Supplementary material for: Performance of PREMM5, clinical criteria, and immunohistochemistry for MMR proteins in genetic risk assessment of Mexican patients with colorectal cancer
Source: PLoS One. 2026 Aug 4;21(8):e0354795. doi: 10.1371/journal.pone.0354795 (PMC13436770; doi:10.1371/journal.pone.0354795)
Supplement: Supplementary file 1 [file pone.0354795.s001.docx]

**S1 Table.** Adenomatous Polyposis Testing Criteria (NCCN Guidelines®). For this study, patients who fulfilled any of these criteria were excluded.

Recommend testing if a personal history of ≥1 of the following criteria:

- ≥20 cumulative adenomas
- Known PV in *APC* in family
- Multifocal/bilateral congenital hypertrophy of retinal pigment epithelium (CHRPE)
- Cribriform-morular variant of papillary thyroid cancer
- Family history of polyposis and family unwilling/unable to have testing

Consider testing if a personal history of ≥1 of the following criteria:

- Between 10–19 cumulative adenomas
- Desmoid tumor
- Hepatoblastoma
- Unilateral CHRPE

**S2 Table.** Family history-based criteria and clinical prediction models performance.

| Criteria / Prediction model | Study Population  N=208 (%) | Non carriers  n=141 (%) | LS genes  n=52 (%) | Other genes  n=15 (%) |
| --- | --- | --- | --- | --- |
| Amsterdam II |  |  |  |  |
| Yes | 41 (19.7) | 11 (7.8) | 28 (53.8) | 2 (13.3) |
| No | 158 (76.0) | 124 (87.9) | 22 (42.3) | 12 (80.0) |
| Non informative | 9 (4.3) | 6 (4.2) | 2 (3.8) | 1 (6.7) |
| Revised Bethesda |  |  |  |  |
| Yes | 178 (85.6) | 113 (80.1) | 51 (98.0) | 14 (93.3) |
| No | 30 (14.4) | 28 (19.9) | 1 (2.0) | 1 (6.7) |
| PREMM5 ≥2.5% |  |  |  |  |
| Yes | 166 (79.8) | 107 (75.9) | 46 (88.5) | 13 (86.6) |
| No  Not estimated | 33 (15.9)  9 (4.3) | 28 (19.9)  6 (4.2) | 4 (7.7)  2 (3.8) | 1 (6.7)  1 (6.7) |
| Median PREMM5 score (range) | 7.1  (0.9 – 50.0) | 5.9  (0.9 – 50.0) | 28.5  (2.1 – 50.0) | 6.4  (2.2 – 29.3) |

MMR genes include: *MLH1*, *PMS2*, *MSH2*, and *MSH6*.

**S3 Table**. Differences between patients with known versus unknown MMR status as determined by immunohistochemistry.

| Characteristic | Known MMR status  N=107 (%) | Unknown MMR status  N=101 (%) | P |
| --- | --- | --- | --- |
| Sex  Female  Male | 58 (54.2)  49 (45.8) | 51 (59.5)  50 (49.5) | 0.677 |
| Median age at CRC diagnosis (Range) | 46.0  (21.0 – 78.0) | 45.0  (18.0 – 82.0) | 0.374 |
| Germline PV  Yes  No | 40 (37.4)  67 (62.6) | 27 (26.7)  74 (73.3) | 0.105 |
| CRC site  Right  Left  Rectum  Unknown | 57 (53.3)  39 (36.4)  8 (7.5)  3 (2.8) | 35 (34.6)  41 (40.6)  21 (20.8)  4 (3.9) | 0.008 |
| Stage  I  II  III  IV  Unavailable | 19 (17.8)  38 (35.5)  28 (26.2)  22 (20.1)  0 (0.0) | 8 (7.9)  15 (14.9)  17 (16.8)  17 (16.8)  44 (43.6) | 0.115 |
