# Supplementary material for: Performance of PREMM5, clinical criteria, and immunohistochemistry for MMR proteins in genetic risk assessment of Mexican patients with colorectal cancer
Source: PLoS One. 2026 Aug 4;21(8):e0354795. doi: 10.1371/journal.pone.0354795 (PMC13436770; doi:10.1371/journal.pone.0354795)
Supplement: Supplementary file 2 [file pone.0354795.s002.docx]

Data Dictionary / Codebook

| Variable | Description | Type | Coding | Units |
| --- | --- | --- | --- | --- |
| sex | Biological sex of the patient | Categorical (nominal) | 1= Male  2= Female | - |
| CRC_site | Primary tumor location | Categorical (nominal) | 0=Unknown  1=Right  2=Left  3=Rectum | _ |
| stage_AJCC_8_ed | Tumor stage according to the American Joint Committee on Cancer (AJCC), 8th edition | Categorical (ordinal) | 0 = Unknown  1 = Stage I  2 = Stage II  3 = Stage III  4 = Stage IV | _ |
| histologic_grade | Histologic tumor grade based on degree of differentiation | Categorical (ordinal) | 0 = Unknown  1 = Well differentiated (G1)  2 = Moderately differentiated (G2)  3 = Poorly differentiated (G3) | - |
| MLH1 | MLH1 protein expression by immunohistochemistry (IHC) | Categorical (nominal) | 0 = Loss of expression  1 = Retained expression  2 = Unknown | - |
| PMS2 | PMS2 protein expression by IHC | Categorical (nominal) | 0 = Loss of expression  1 = Retained expression  2 = Unknown | - |
| MSH2 | MSH2 protein expression by IHC | Categorical (nominal) | 0 = Loss of expression  1 = Retained expression  2 = Unknown | - |
| MSH6 | MSH6 protein expression by IHC | Categorical (nominal) | 0 = Loss of expression  1 = Retained expression  2 = Unknown | - |
| dMMR_status | Mismatch repair deficiency status based on immunohistochemistry (MLH1, MSH2, MSH6, PMS2) | Categorical (nominal) | 1 = deficient  2 = proficient  3 = unknown | - |
| IHC_concordance | Concordance between IHC results and germline genetic testing | Categorical (nominal) | 1 = yes  2 = no  3 = not assessed | - |
| germline_genetic_testing_result | Result of germline genetic testing for hereditary cancer predisposition genes | Categorical (nominal) | Negative or detection of a pathogenic or likely pathogenic variant | - |
| second_primary | Presence of a second primary malignancy distinct from the index colorectal cancer | Categorical (nominal) | Site of second primary malignancy | - |
| age_2 | Age at diagnosis of the second primary malignancy | Continuous | Numeric | years |
| third_primary | Presence of a third primary malignancy distinct from the index colorectal cancer | Categorical (nominal) | Site of third primary malignancy | - |
| age_3 | Age at diagnosis of the third primary malignancy | Continuous | Numeric | years |
| second_CRC_timing | Timing of a second primary colorectal cancer relative to the index tumor | Categorical (nominal) | 0 = No second colorectal cancer  1 = Synchronous  2 = Metachronous | - |
| family_history_crc | Number of first- and second-degree relatives with colorectal cancer | Continuous | Numeric | Family members |
| family_history_endometrial | Number of first- and second-degree relatives with endometrial cancer | Continuous | Numeric | Family members |
| family_history_gastric | Number of first- and second-degree relatives with gastric cancer | Continuous | Numeric | Family members |
| family_history_small_intestine | Number of first- and second-degree relatives with small intestine cancer | Continuous | Numeric | Family members |
| family_history_pancreatic | Number of first- and second-degree relatives with pancreatic cancer | Continuous | Numeric | Family members |
| family_history_biliary_tract | Number of first- and second-degree relatives with biliary tract cancer | Continuous | Numeric | Family members |
| family_history_urinary_tract_bladder_kidney | Number of first- and second-degree relatives with urinary tract / bladder / kidney cancer | Continuous | Numeric | Family members |
| family_history_ovarian | Number of first- and second-degree relatives with ovarian cancer | Continuous | Numeric | Family members |
| family_history_sebaceous_gland | Number of first- and second-degree relatives with sebaceous gland cancer | Continuous | Numeric | Family members |
| family_history_cns | Number of first- and second-degree relatives with central nervous system cancer | Continuous | Numeric | Family members |
| lynch_syndrome_diagnosis | Diagnosis of Lynch syndrome based on germline genetic testing | Categorical (binary) | 0 = No  1 = Yes | - |
| premm5 | Predicted probability of carrying a germline pathogenic variant in mismatch repair genes, estimated using the PREMM5 model | Continuous | Numeric | Percentage (%) |
| amsterdam_II | Fulfillment of the Amsterdam II criteria for Lynch syndrome | Categorical (binary) | 0 = No  1 = Yes | - |
| bethesda | Fulfillment of the revised Bethesda criteria | Categorical (binary) | 0 = No  1 = Yes | - |
