# Supplementary material for: Performance of PREMM5, clinical criteria, and immunohistochemistry for MMR proteins in genetic risk assessment of Mexican patients with colorectal cancer
Source: PLoS One. 2026 Aug 4;21(8):e0354795. doi: 10.1371/journal.pone.0354795 (PMC13436770; doi:10.1371/journal.pone.0354795)
Supplement: Supplementary file 3 [file pone.0354795.s005.docx]

May 5, 2026

**Dr. Albert Rübben, MD, PhD**

**Academic Editor PLOS One**

We would like to thank “PLOS One” for considering our paper. We also thank the reviewers for their hard work and helpful comments, which certainly contributed to improve the overall quality of the manuscript. Enclosed please find the revised manuscript and a detailed point-by-point description addressing the editor’s and reviewers’ comments. All authors have read and approved the final version of this manuscript and concur with the submission.

Sincerely,

**Yanin Chavarri-Guerra, MD, PhD.**

**Corresponding author**

**Reviewer #1**

Dear Editor, I have reviewed the manuscript evaluating the performance of PREMM5, clinical criteria (Amsterdam II and revised Bethesda), and mismatch repair (MMR) immunohistochemistry for identifying germline pathogenic variants in Mexican patients with colorectal cancer. This study addresses an important gap by assessing genetic risk assessment tools in an underrepresented Hispanic/Mestizo population. The multicenter design, use of multigene panel testing, and focus on resource-limited clinical settings are notable strengths. The reported diagnostic performance of PREMM5 (AUC ≈0.82) and the high proportion of Lynch syndrome cases provide clinically relevant regional data. However, several issues related to methodological transparency, potential bias, and reporting must be addressed before the manuscript is suitable for publication.

R= We thank the reviewers for their thoughtful and constructive comments. We have carefully addressed all concerns and revised the manuscript accordingly to improve methodological transparency, clarity, and interpretation of our findings. All changes are indicated in the revised manuscript.

Major concerns

1. Selection bias and generalizability. The cohort consists exclusively of patients referred for genetic cancer risk assessment based on clinical criteria, abnormal IHC, or elevated PREMM5 scores, and is characterized by early age at diagnosis and a high prevalence of dMMR tumors. This referral-enriched design likely explains the high pathogenic variant yield (32.2%) and may inflate diagnostic performance estimates. The authors should clearly state that the findings are not generalizable to unselected colorectal cancer populations and temper conclusions accordingly.

R= We thank the reviewer for this important observation. We acknowledge that our cohort represents a highly selected population, which likely enriched the prevalence of germline pathogenic variants and may overestimate diagnostic performance. We have clarified this in the Methods and Discussion sections and have tempered our conclusions accordingly. Specifically, we now explicitly state that these findings are not generalized to unselected colorectal cancer populations and should interpreted within the context of a referral-enriched cohort.

2. Missing IHC data. IHC results were available for only about half of the cohort. The reasons for missing data are not described, and potential verification or selection bias is not discussed. The authors should report why IHC was unavailable and compare characteristics of patients with and without IHC results.

R= We agree with the reviewer that incomplete IHC data may introduce selection and verification bias. We have now clarified those reasons for missing IHC in the Methods, including limited tissue availability and variability in institutional practices across referring institutions. In addition, we performed a comparison of baseline clinical and pathologic characteristics between patients with and without available IHC results (Appendix C) to assess potential bias: No statistical differences were observed between groups. This limitation and its potential impact on interpretation of our findings are now also acknowledged in the Discussion.

3. Statistical reporting clarity. The confidence interval for the PREMM5 AUC appears unusually narrow and should be verified. In addition, the primary outcome (Lynch syndrome vs. any germline pathogenic variant) should be clearly defined and used consistently throughout the manuscript.

R= We thank the reviewer for this important observation. We identified an error in the original that led to an incorrect display of the confidence interval bounds. The ROC analysis has been rerun and the figure corrected. The updated AUC for PREMM5 is 0.822 (95% CI: 0.746–0.898, DeLong method), consistent with the originally reported point estimate.

In addition, we have clarified and standardized throughout the manuscript that the primary outcome for diagnostic performance analyses is the identification of Lynch syndrome (germline pathogenic variants in MMR genes).

4. Data availability. The current statement indicating that data are available upon request does not comply with PLOS ONE data sharing requirements. The dataset should be deposited in a public repository or an appropriate controlled-access justification provided.

R= We thank the reviewer for this important comment and fully agree with the principles of open data transparency.

The dataset underlying the findings of this study has now been made publicly available as a supplementary file. All direct identifiers have been removed, and genetic data have been limited to the gene level (without specific variant information) to minimize the risk of participant re-identification.

The Data Availability statement has been updated accordingly.

5. Interpretation of non-Lynch gene findings. Approximately 22% of pathogenic variants were identified in non-Lynch genes. Given the variable and sometimes uncertain colorectal cancer risk associated with several of these genes, the recommendation for multigene testing should be presented more cautiously and with clearer discussion of clinical actionability.

R= We agree with the reviewer that the clinical implications of pathogenic variants in non-Lynch genes are variable and, in some cases, uncertain. We have revised the Discussion to provide a more cautious interpretation, emphasizing current guideline recommendations and the limited colorectal cancer risk associated with several of these genes. We have also clarified that the identification of such variants highlights the complexity of multigene panel testing rather than supporting universal expansion without careful clinical interpretation.

Minor comments

- Improve grammatical consistency and reduce redundancy in the Introduction. • Clarify technical details of sequencing and CNV detection across genes.
- Report missing percentage symbols and formatting inconsistencies in tables (e.g.,

Table	3).

- Consider adding a participant flow diagram to improve transparency.
- The limitations section should explicitly acknowledge referral bias, missing data, and limited generalizability.

Conclusion

This is a clinically relevant and potentially valuable study that contributes important data from an underrepresented population. However, clarification of cohort selection, improved transparency regarding missing data and statistical reporting, compliance with data availability requirements, and more cautious interpretation are necessary. I recommend major revision prior to further consideration.

R= We have revised the manuscript to improve grammatical consistency and reduce redundancy in the Introduction. Technical details of sequencing and copy-number variant detection have been clarified. Formatting inconsistencies in tables have been corrected, and missing percentage symbols have been added. A participant flow diagram has been incorporated as Figure 1. Finally, the limitations section has been expanded to explicitly address referral bias, missing data, and limited generalizability.

**Reviewer #2**

The authors present an analysis of multiple screening strategies as applied to patients with colorectal cancer for the purpose of identifying those at increased likelihood of being identified on germline genetic testing as having Lynch syndrome, or a germline P/LP conferring other syndromic genetic cancer risk. The strategies analyzed here have been previously validated. This analysis is somewhat novel in its focus on a cohort of predominantly Mexican descent and presents data potentially useful to the CRC clinical community. Prior to being considered further, I recommend the follow comments/questions be addressed.

R= We sincerely thank the reviewer for these thoughtful comments, which greatly improved the clarity and interpretation of our study methodology. In response, we have revised the manuscript to explicitly describe the initial screening phase used to identify eligible participants. This distinction is essential, as our cohort was designed to represent patients prioritized for germline genetic testing in a resource-limited setting rather than an unselected colorectal cancer population. We believe that this clarification provides a more accurate methodological context for interpreting the performance of the evaluated tools and better reflects current real-world clinical practice in our setting.

1. In the Introduction the term "Genetic Cancer Risk Assessment" or GCRA is introduced, however what this actually is does not appear to be explained and needs to be defined. Is GCRA the clinical intake whereby patient personal and family health history are document? Is it the process whereby collected patient clinical information is reviewed for whether it meets guidelines for genetic testing? Is the GCRA the process by which patients are compared against Amsterdam, Bethesda, PREMM5 or IHC to assess whether they meet these criteria for germline testing? Does it include the process of germline genetic testing to assess the future cancer risk of a patient with CRC? Please clarify.

R= We thank the reviewer for highlighting the need for clarification. We have now defined Genetic Cancer Risk Assessment (GCRA) in the Introduction as a

comprehensive clinical process that includes detailed personal and family history assessment, risk stratification using clinical criteria and prediction models, and evaluation for germline genetic testing when indicated.

2. In the Introduction is stated "GCRA is considered a standard of care for selected patients with colorectal cancer". Depending on the answer to the above re: GCRA, it is the standard of care for all patients with CRC to have their cancer risk assessed by collection, analysis and comparison of their clinical information to confirm their eligibility for germline testing. NCCN guidelines indicate germline testing should be considered in all patients with CRC. If the primary intent of this work is to validate PREMM5 as a strategy to be applied in resource constrained geographies for selectively allotting germline testing only to those patients at predicted higher relative risk for having a P/LP, this should be clarified at the outset.

R= We appreciate this important clarification. We have revised the statement to reflect current guidelines, acknowledging that germline testing should be considered in all patients with colorectal cancer. We further clarify that, in resource-constrained settings, tools such as PREMM5 may help prioritize patients for testing when universal access is not feasible.

3. In the Methods the inclusion criteria are outlined, e.g. >=18 yo, diagnosis of CRC, age at diagnosis <50yo, etc. These inclusion criteria represent a "pre-screen" of patients with CRC which has implications for the total number/percentage of patients with P/LP identified after subsequent application of the screening strategies applied. The fact that these inclusion criteria functioned to pre-screen the total population of patients with CRC at the two participating institutions and select only certain qualifying CRC patients for the study needs to be explained in the text. Please add this to the Methods.

R= We agree with the reviewer that the inclusion criteria effectively functioned as a pre-screening step. We have now clarified this in the Methods section, explicitly stating that only patients meeting predefined clinical criteria were invited to participate, which enriched the study population for hereditary cancer risk.

4. To the comment above, how many patients with CRC were "pre-screened" using the inclusion criteria in order to obtain the 208 patients that were enrolled? i.e. what was the total population of patients with CRC seen at these two institutions during the enrollment timeframe of the study?

R= During the study period, a total of 1,081 patients with colorectal cancer were evaluated at the participating institutions, of whom 208 met the inclusion criteria and completed all study procedures. This information has now been added to the Methods section. This process is also illustrated in the newly added Figure 1 as a flowchart.

5. Methods paragraph 3 is noted patients were consented to the study then underwent GCRA. Was prescreening using inclusion criteria performed before or after obtaining consent, or were only those patients who met the inclusion criteria invited to give their consent to be part of the study? Here again, there is a need to define what it means that patients "underwent GCRA". Please clarify.

R= We have clarified that pre-screening based on clinical criteria was performed prior to obtaining informed consent, and only eligible patients were invited to participate.

6. Methods section paragraph 4 notes lack of genetic testing coverage for *PMS2* exons 11-15 and *PTEN* promoter region. This should be reiterated in the Discussion as a limitation as it impacts interpretation of the overall P/LP prevalence in the tested population with some studies suggesting >10% of *PMS2* P/LP occurring in exons 11-5.

R= We agree and have now included this in the Limitations section, acknowledging that incomplete coverage of *PMS2* exons 11–15 and the *PTEN* promoter region may have led to underdetection of pathogenic variants; this statement is now supported by two additional references.

7. Also in Methods paragraph 4, second to last line indicates "...*MSH2* and *MSH6* were also analyzed for number variants...", where I believe "copy-number variants" is intended, please correct if so.

R= We thank the reviewer for noting this. The term has been corrected to “copynumber variants.”

8. Discussion section, first line states "individuals with CRC who met clinical criteria for GCRA". Again, what does GCRA mean? and what are the clinical criteria that were met for a patient to undergo GCRA? This needs to be clarified as it is currently unclear if the clinical criteria are the inclusion criteria, the criteria represented by each of the screening strategies (PREMM5, Amsterdam, Bethesda, IHC), NCCN criteria or something else.

R= We have revised the Discussion to ensure consistent and clear use of the term GCRA, explicitly referring to the predefined inclusion criteria and clinical risk assessment process used in this study.

9. Discussion section, second paragraph, "...77.6% of hereditary CRC cases were attributed to Lynch syndrome..." should be changed to, "77.6% of selected hereditary CRC cases...", as all patients were prescreened prior to enrollment and all enrolled patients were screened via PREMM5, Amsterdam, Bethesda or IHC prior to germline testing.

R= We agree and have revised the text to read “selected hereditary CRC cases” to reflect the pre-screened nature of the cohort.
