# Supplementary material for: Performance of PREMM5, clinical criteria, and immunohistochemistry for MMR proteins in genetic risk assessment of Mexican patients with colorectal cancer
Source: PLoS One. 2026 Aug 4;21(8):e0354795. doi: 10.1371/journal.pone.0354795 (PMC13436770; doi:10.1371/journal.pone.0354795)
Supplement: Peer review history

# Peer review: Decision Letter 0

**DOI:** 10.1371/journal.pone.0354795.r001

**Authors:** Albert Rübben

10 Mar 2026

PONE-D-25-45234

Performance of PREMM5, Clinical Criteria, and Immunohistochemistry for MMR Proteins in Genetic Risk Assessment of Mexican patients with Colorectal Cancer

PLOS One

Dear Dr. Chávarri-Guerra,

Thank you for submitting your manuscript to PLOS ONE. After careful consideration, we feel that it has merit but does not fully meet PLOS ONE’s publication criteria as it currently stands. Therefore, we invite you to submit a revised version of the manuscript that addresses the points raised during the review process.

Please submit your revised manuscript by Apr 24 2026 11:59PM. If you will need more time than this to complete your revisions, please reply to this message or contact the journal office at plosone@plos.org. When you're ready to submit your revision, log on to https://www.editorialmanager.com/pone/ and select the 'Submissions Needing Revision' folder to locate your manuscript file.

Please include the following items when submitting your revised manuscript:

- A letter that responds to each point raised by the academic editor and reviewer(s). You should upload this letter as a separate file labeled 'Response to Reviewers'.
- A marked-up copy of your manuscript that highlights changes made to the original version. You should upload this as a separate file labeled 'Revised Manuscript with Track Changes'.
- An unmarked version of your revised paper without tracked changes. You should upload this as a separate file labeled 'Manuscript'.

If you would like to make changes to your financial disclosure, please include your updated statement in your cover letter. Guidelines for resubmitting your figure files are available below the reviewer comments at the end of this letter.

If applicable, we recommend that you deposit your laboratory protocols in protocols.io to enhance the reproducibility of your results. Protocols.io assigns your protocol its own identifier (DOI) so that it can be cited independently in the future. For instructions see: https://journals.plos.org/plosone/s/submission-guidelines#loc-laboratory-protocols. Additionally, PLOS ONE offers an option for publishing peer-reviewed Lab Protocol articles, which describe protocols hosted on protocols.io. Read more information on sharing protocols at https://plos.org/protocols?utm_medium=editorial-email&utm_source=authorletters&utm_campaign=protocols.

We look forward to receiving your revised manuscript.

Kind regards,

Albert Rübben, Ass. Prof., M.D., Ph.D.

Academic Editor

PLOS One

Journal Requirements:

When submitting your revision, we need you to address these additional requirements.

1.Please ensure that your manuscript meets PLOS ONE's style requirements, including those for file naming. The PLOS ONE style templates can be found at

https://journals.plos.org/plosone/s/file?id=wjVg/PLOSOne_formatting_sample_main_body.pdf and

https://journals.plos.org/plosone/s/file?id=ba62/PLOSOne_formatting_sample_title_authors_affiliations.pdf

2. PLOS requires an ORCID iD for the corresponding author in Editorial Manager on papers submitted after December 6th, 2016. Please ensure that you have an ORCID iD and that it is validated in Editorial Manager. To do this, go to ‘Update my Information’ (in the upper left-hand corner of the main menu), and click on the Fetch/Validate link next to the ORCID field. This will take you to the ORCID site and allow you to create a new iD or authenticate a pre-existing iD in Editorial Manager.

3. Please include your tables as part of your main manuscript and remove the individual files. Please note that supplementary tables (should remain/ be uploaded) as separate "supporting information" files.

4. Thank you for stating the following financial disclosure:

“Some sequencing was performed through the GRACIAS project, with support from the Breast Cancer Research Foundation grant 24-210, and the Conquer Cancer Research Professorship in Breast Cancer Disparities (J.N. Weitzel).”

Please state what role the funders took in the study.  If the funders had no role, please state: "The funders had no role in study design, data collection and analysis, decision to publish, or preparation of the manuscript."

If this statement is not correct you must amend it as needed.

Please include this amended Role of Funder statement in your cover letter; we will change the online submission form on your behalf.

5. Thank you for stating the following in the Competing Interests section:

“J.N. Weitzel reports consulting fees for Natera, MyOme and Cancer IQ. Y. Chávarri reports research support from Roche and Pfizer; Speaker´s bureau for AstraZeneca, Gilead, Novartis, Roche and Lilly; travel expenses for Novartis, Pfizer, Lilly and Gilead. All other authors reported no disclosures.”

We note that one or more of the authors are employed by a commercial company: “Roche and Pfizer, Novartis, Pfizer, Lilly and Gilead”

1. Please provide an amended Funding Statement declaring this commercial affiliation, as well as a statement regarding the Role of Funders in your study. If the funding organization did not play a role in the study design, data collection and analysis, decision to publish, or preparation of the manuscript and only provided financial support in the form of authors' salaries and/or research materials, please review your statements relating to the author contributions, and ensure you have specifically and accurately indicated the role(s) that these authors had in your study. You can update author roles in the Author Contributions section of the online submission form.

Please also include the following statement within your amended Funding Statement.

“The funder provided support in the form of salaries for authors “J.N.W. and Y.C.” but did not have any additional role in the study design, data collection and analysis, decision to publish, or preparation of the manuscript. The specific roles of these authors are articulated in the ‘author contributions’ section.”

If your commercial affiliation did play a role in your study, please state and explain this role within your updated Funding Statement.

2. Please also provide an updated Competing Interests Statement declaring this commercial affiliation along with any other relevant declarations relating to employment, consultancy, patents, products in development, or marketed products, etc.

Within your Competing Interests Statement, please confirm that this commercial affiliation does not alter your adherence to all PLOS ONE policies on sharing data and materials by including the following statement: "This does not alter our adherence to  PLOS ONE policies on sharing data and materials.” (as detailed online in our guide for authors http://journals.plos.org/plosone/s/competing-interests) . If this adherence statement is not accurate and  there are restrictions on sharing of data and/or materials, please state these. Please note that we cannot proceed with consideration of your article until this information has been declared.

Please include both an updated Funding Statement and Competing Interests Statement in your cover letter. We will change the online submission form on your behalf.

6. In the online submission form, you indicated that your data is available only on request from a third party. Please note that your Data Availability Statement is currently missing the name of the third party contact or institution / contact details for the third party, such as an email address or a link to where data requests can be made. Please update your statement with the missing information.

7. We note you have included a table to which you do not refer in the text of your manuscript. Please ensure that you refer to Table 3 in your text; if accepted, production will need this reference to link the reader to the Table.

8. If the reviewer comments include a recommendation to cite specific previously published works, please review and evaluate these publications to determine whether they are relevant and should be cited. There is no requirement to cite these works unless the editor has indicated otherwise.

Additional Editor Comments:

Both reviewers raise concerns regarding potential selection bias due to pre-screening/referral enrichment, which should be clearly described and its implications addressed prior to further consideration. Please also address the reviewers’ comments regarding incomplete IHC testing data and clarify/verify the statistical analyses and reporting.

[Note: HTML markup is below. Please do not edit.]

Reviewer's Responses to Questions

**Comments to the Author**

1. Is the manuscript technically sound, and do the data support the conclusions?

The manuscript must describe a technically sound piece of scientific research with data that supports the conclusions. Experiments must have been conducted rigorously, with appropriate controls, replication, and sample sizes. The conclusions must be drawn appropriately based on the data presented.

Reviewer #1: Yes

Reviewer #2: Yes

**********

2. Has the statistical analysis been performed appropriately and rigorously?

Reviewer #1: Yes

Reviewer #2: Yes

**********

3. Have the authors made all data underlying the findings in their manuscript fully available?

The PLOS Data policy requires authors to make all data underlying the findings described in their manuscript fully available without restriction, with rare exception (please refer to the Data Availability Statement in the manuscript PDF file). The data should be provided as part of the manuscript or its supporting information, or deposited to a public repository. For example, in addition to summary statistics, the data points behind means, medians and variance measures should be available. If there are restrictions on publicly sharing data—e.g. participant privacy or use of data from a third party—those must be specified.

Reviewer #1: Yes

Reviewer #2: Yes

**********

4. Is the manuscript presented in an intelligible fashion and written in standard English?

PLOS ONE does not copyedit accepted manuscripts, so the language in submitted articles must be clear, correct, and unambiguous. Any typographical or grammatical errors should be corrected at revision, so please note any specific errors here.

Reviewer #1: Yes

Reviewer #2: Yes

**********

5. Review Comments to the Author

Please use the space provided to explain your answers to the questions above. You may also include additional comments for the author, including concerns about dual publication, research ethics, or publication ethics. (Please upload your review as an attachment if it exceeds 20,000 characters)

Reviewer #1: Dear Editor,

I have reviewed the manuscript evaluating the performance of PREMM5, clinical criteria (Amsterdam II and revised Bethesda), and mismatch repair (MMR) immunohistochemistry for identifying germline pathogenic variants in Mexican patients with colorectal cancer. This study addresses an important gap by assessing genetic risk assessment tools in an underrepresented Hispanic/Mestizo population. The multicenter design, use of multigene panel testing, and focus on resource-limited clinical settings are notable strengths. The reported diagnostic performance of PREMM5 (AUC ≈0.82) and the high proportion of Lynch syndrome cases provide clinically relevant regional data.

However, several issues related to methodological transparency, potential bias, and reporting must be addressed before the manuscript is suitable for publication.

Major concerns

1. Selection bias and generalizability

The cohort consists exclusively of patients referred for genetic cancer risk assessment based on clinical criteria, abnormal IHC, or elevated PREMM5 scores, and is characterized by early age at diagnosis and a high prevalence of dMMR tumors. This referral-enriched design likely explains the high pathogenic variant yield (32.2%) and may inflate diagnostic performance estimates. The authors should clearly state that the findings are not generalizable to unselected colorectal cancer populations and temper conclusions accordingly.

2. Missing IHC data

IHC results were available for only about half of the cohort. The reasons for missing data are not described, and potential verification or selection bias is not discussed. The authors should report why IHC was unavailable and compare characteristics of patients with and without IHC results.

3. Statistical reporting clarity

The confidence interval for the PREMM5 AUC appears unusually narrow and should be verified. In addition, the primary outcome (Lynch syndrome vs. any germline pathogenic variant) should be clearly defined and used consistently throughout the manuscript.

4. Data availability

The current statement indicating that data are available upon request does not comply with PLOS ONE data sharing requirements. The dataset should be deposited in a public repository or an appropriate controlled-access justification provided.

5. Interpretation of non-Lynch gene findings

Approximately 22% of pathogenic variants were identified in non-Lynch genes. Given the variable and sometimes uncertain colorectal cancer risk associated with several of these genes, the recommendation for multigene testing should be presented more cautiously and with clearer discussion of clinical actionability.

Minor comments

• Improve grammatical consistency and reduce redundancy in the Introduction.

• Clarify technical details of sequencing and CNV detection across genes.

• Report missing percentage symbols and formatting inconsistencies in tables (e.g., Table 3).

• Consider adding a participant flow diagram to improve transparency.

• The limitations section should explicitly acknowledge referral bias, missing data, and limited generalizability.

Conclusion

This is a clinically relevant and potentially valuable study that contributes important data from an underrepresented population. However, clarification of cohort selection, improved transparency regarding missing data and statistical reporting, compliance with data availability requirements, and more cautious interpretation are necessary. I recommend major revision prior to further consideration.

Reviewer #2: The authors present an analysis of multiple screening strategies as applied to patients with colorectal cancer for the purpose of identifying those at increased likelihood of being identified on germline genetic testing as having Lynch syndrome, or a germline P/LP conferring other syndromic genetic cancer risk. The strategies analyzed here have been previously validated. This analysis is somewhat novel in its focus on a cohort of predominantly Mexican descent and presents data potentially useful to the CRC clinical community. Prior to being considered further, I recommend the follow comments/questions be addressed:

1 - in the Introduction the term "Genetic Cancer Risk Assessment" or GCRA is introduced, however what this actually is does not appear to be explained and needs to be defined. Is GCRA the clinical intake whereby patient personal and family health history are document? Is it the process whereby collected patient clinical information is reviewed for whether it meets guidelines for genetic testing? Is the GCRA the process by which patients are compared against Amsterdam, Bethesda, PREMM5 or IHC to assess whether they meet these criteria for germline testing? Does it include the process of germline genetic testing to assess the future cancer risk of a patient with CRC? Please clarify.

2 - In the Introduction is stated "GCRA is considered a standard of care for selected patients with colorectal cancer". Depending on the answer to the above re: GCRA, it is the standard of care for all patients with CRC to have their cancer risk assessed by collection, analysis and comparison of their clinical information to confirm their eligibility for germline testing. NCCN guidelines indicate germline testing should be considered in all patients with CRC. If the primary intent of this work is to validate PREMM5 as a strategy to be applied in resource constrained geographies for selectively allotting germline testing only to those patients at predicted higher relative risk for having a P/LP, this should be clarified at the outset.

3 - In the Methods the inclusion criteria are outlined, e.g. >=18 yo, diagnosis of CRC, age at diagnosis <50yo, etc. These inclusion criteria represent a "pre-screen" of patients with CRC which has implications for the total number/percentage of patients with P/LP identified after subsequent application of the screening strategies applied. The fact that these inclusion criteria functioned to pre-screen the total population of patients with CRC at the two participating institutions and select only certain qualifying CRC patients for the study needs to be explained in the text. Please add this to the Methods.

4 - To the comment above, how many patients with CRC were "pre-screened" using the inclusion criteria in order to obtain the 208 patients that were enrolled? i.e. what was the total population of patients with CRC seen at these two institutions during the enrollment timeframe of the study?

5 - Methods paragraph 3 is noted patients were consented to the study then underwent GCRA. Was prescreening using inclusion criteria performed before or after obtaining consent, or were only those patients who met the inclusion criteria invited to give their consent to be part of the study? Here again, there is a need to define what it means that patients "underwent GCRA". Please clarify.

6 - Methods section paragraph 4 notes lack of genetic testing coverage for PMS2 exons 11-15 and PTEN promoter region. This should be reiterated in the Discussion as a limitation as it impacts interpretation of the overall P/LP prevalence in the tested population with some studies suggesting >10% of PMS2 P/LP occurring in exons 11-5.

7 - Also in Methods paragraph 4, second to last line indicates "...MSH2 and MSH6 were also analyzed for number variants...", where I believe "copy-number variants" is intended, please correct if so.

8 - Discussion section, first line states "individuals with CRC who met clinical criteria for GCRA". Again, what does GCRA mean? and what are the clinical criteria that were met for a patient to undergo GCRA? This needs to be clarified as it is currently unclear if the clinical criteria are the inclusion criteria, the criteria represented by each of the screening strategies (PREMM5, Amsterdam, Bethesda, IHC), NCCN criteria or something else.

9 - Discussion section, second paragraph, "...77.6% of hereditary CRC cases were attributed to Lynch syndrome..." should be changed to, "77.6% of selected hereditary CRC cases...", as all patients were prescreened prior to enrollment and all enrolled patients were screened via PREMM5, Amsterdam, Bethesda or IHC prior to germline testing.

**********

6. PLOS authors have the option to publish the peer review history of their article (what does this mean?). If published, this will include your full peer review and any attached files.

If you choose “no”, your identity will remain anonymous but your review may still be made public.

**Do you want your identity to be public for this peer review?**  For information about this choice, including consent withdrawal, please see our Privacy Policy.

Reviewer #1: **Yes:** Mahmut Cerkez Ergoren

Reviewer #2: **Yes:** Edward Esplin

**********

[NOTE: If reviewer comments were submitted as an attachment file, they will be attached to this email and accessible via the submission site. Please log into your account, locate the manuscript record, and check for the action link "View Attachments". If this link does not appear, there are no attachment files.]

To ensure your figures meet our technical requirements, please review our figure guidelines: https://journals.plos.org/plosone/s/figures

You may also use PLOS’s free figure tool, NAAS, to help you prepare publication quality figures: https://journals.plos.org/plosone/s/figures#loc-tools-for-figure-preparation.

NAAS will assess whether your figures meet our technical requirements by comparing each figure against our figure specifications.

# Author comment: Author response to Decision Letter 1

**DOI:** 10.1371/journal.pone.0354795.r002

9 May 2026

Reviewer #1

Dear Editor, I have reviewed the manuscript evaluating the performance of PREMM5, clinical criteria (Amsterdam II and revised Bethesda), and mismatch repair (MMR) immunohistochemistry for identifying germline pathogenic variants in Mexican patients with colorectal cancer. This study addresses an important gap by assessing genetic risk assessment tools in an underrepresented Hispanic/Mestizo population. The multicenter design, use of multigene panel testing, and focus on resource-limited clinical settings are notable strengths. The reported diagnostic performance of PREMM5 (AUC ≈0.82) and the high proportion of Lynch syndrome cases provide clinically relevant regional data. However, several issues related to methodological transparency, potential bias, and reporting must be addressed before the manuscript is suitable for publication.

R= We thank the reviewers for their thoughtful and constructive comments. We have carefully addressed all concerns and revised the manuscript accordingly to improve methodological transparency, clarity, and interpretation of our findings. All changes are indicated in the revised manuscript.

Major concerns

1. Selection bias and generalizability. The cohort consists exclusively of patients referred for genetic cancer risk assessment based on clinical criteria, abnormal IHC, or elevated PREMM5 scores, and is characterized by early age at diagnosis and a high prevalence of dMMR tumors. This referral-enriched design likely explains the high pathogenic variant yield (32.2%) and may inflate diagnostic performance estimates. The authors should clearly state that the findings are not generalizable to unselected colorectal cancer populations and temper conclusions accordingly.

R= We thank the reviewer for this important observation. We acknowledge that our cohort represents a highly selected population, which likely enriched the prevalence of germline pathogenic variants and may overestimate diagnostic performance. We have clarified this in the Methods and Discussion sections and have tempered our conclusions accordingly. Specifically, we now explicitly state that these findings are not generalized to unselected colorectal cancer populations and should interpreted within the context of a referral-enriched cohort.

2. Missing IHC data. IHC results were available for only about half of the cohort. The reasons for missing data are not described, and potential verification or selection bias is not discussed. The authors should report why IHC was unavailable and compare characteristics of patients with and without IHC results.

R= We agree with the reviewer that incomplete IHC data may introduce selection and verification bias. We have now clarified those reasons for missing IHC in the Methods, including limited tissue availability and variability in institutional practices across referring institutions. In addition, we performed a comparison of baseline clinical and pathologic characteristics between patients with and without available IHC results (Appendix C) to assess potential bias: No statistical differences were observed between groups. This limitation and its potential impact on interpretation of our findings are now also acknowledged in the Discussion.

3. Statistical reporting clarity. The confidence interval for the PREMM5 AUC appears unusually narrow and should be verified. In addition, the primary outcome (Lynch syndrome vs. any germline pathogenic variant) should be clearly defined and used consistently throughout the manuscript.

R= We thank the reviewer for this important observation. We identified an error in the original that led to an incorrect display of the confidence interval bounds. The ROC analysis has been rerun and the figure corrected. The updated AUC for PREMM5 is 0.822 (95% CI: 0.746–0.898, DeLong method), consistent with the originally reported point estimate.

In addition, we have clarified and standardized throughout the manuscript that the primary outcome for diagnostic performance analyses is the identification of Lynch syndrome (germline pathogenic variants in MMR genes).

4. Data availability. The current statement indicating that data are available upon request does not comply with PLOS ONE data sharing requirements. The dataset should be deposited in a public repository or an appropriate controlled-access justification provided.

R= We thank the reviewer for this important comment and fully agree with the principles of open data transparency.

The dataset underlying the findings of this study has now been made publicly available as a supplementary file. All direct identifiers have been removed, and genetic data have been limited to the gene level (without specific variant information) to minimize the risk of participant re-identification.

The Data Availability statement has been updated accordingly.

5. Interpretation of non-Lynch gene findings. Approximately 22% of pathogenic variants were identified in non-Lynch genes. Given the variable and sometimes uncertain colorectal cancer risk associated with several of these genes, the recommendation for multigene testing should be presented more cautiously and with clearer discussion of clinical actionability.

R= We agree with the reviewer that the clinical implications of pathogenic variants in non-Lynch genes are variable and, in some cases, uncertain. We have revised the Discussion to provide a more cautious interpretation, emphasizing current guideline recommendations and the limited colorectal cancer risk associated with several of these genes. We have also clarified that the identification of such variants highlights the complexity of multigene panel testing rather than supporting universal expansion without careful clinical interpretation.

Minor comments

• Improve grammatical consistency and reduce redundancy in the Introduction. • Clarify technical details of sequencing and CNV detection across genes.

• Report missing percentage symbols and formatting inconsistencies in tables (e.g.,

Table 3).

• Consider adding a participant flow diagram to improve transparency.

• The limitations section should explicitly acknowledge referral bias, missing data, and limited generalizability.

Conclusion

This is a clinically relevant and potentially valuable study that contributes important data from an underrepresented population. However, clarification of cohort selection, improved transparency regarding missing data and statistical reporting, compliance with data availability requirements, and more cautious interpretation are necessary. I recommend major revision prior to further consideration.

R= We have revised the manuscript to improve grammatical consistency and reduce redundancy in the Introduction. Technical details of sequencing and copy-number variant detection have been clarified. Formatting inconsistencies in tables have been corrected, and missing percentage symbols have been added. A participant flow diagram has been incorporated as Figure 1. Finally, the limitations section has been expanded to explicitly address referral bias, missing data, and limited generalizability.

Reviewer #2

The authors present an analysis of multiple screening strategies as applied to patients with colorectal cancer for the purpose of identifying those at increased likelihood of being identified on germline genetic testing as having Lynch syndrome, or a germline P/LP conferring other syndromic genetic cancer risk. The strategies analyzed here have been previously validated. This analysis is somewhat novel in its focus on a cohort of predominantly Mexican descent and presents data potentially useful to the CRC clinical community. Prior to being considered further, I recommend the follow comments/questions be addressed.

R= We sincerely thank the reviewer for these thoughtful comments, which greatly improved the clarity and interpretation of our study methodology. In response, we have revised the manuscript to explicitly describe the initial screening phase used to identify eligible participants. This distinction is essential, as our cohort was designed to represent patients prioritized for germline genetic testing in a resource-limited setting rather than an unselected colorectal cancer population. We believe that this clarification provides a more accurate methodological context for interpreting the performance of the evaluated tools and better reflects current real-world clinical practice in our setting.

1. In the Introduction the term "Genetic Cancer Risk Assessment" or GCRA is introduced, however what this actually is does not appear to be explained and needs to be defined. Is GCRA the clinical intake whereby patient personal and family health history are document? Is it the process whereby collected patient clinical information is reviewed for whether it meets guidelines for genetic testing? Is the GCRA the process by which patients are compared against Amsterdam, Bethesda, PREMM5 or IHC to assess whether they meet these criteria for germline testing? Does it include the process of germline genetic testing to assess the future cancer risk of a patient with CRC? Please clarify.

R= We thank the reviewer for highlighting the need for clarification. We have now defined Genetic Cancer Risk Assessment (GCRA) in the Introduction as a

comprehensive clinical process that includes detailed personal and family history assessment, risk stratification using clinical criteria and prediction models, and evaluation for germline genetic testing when indicated.

2. In the Introduction is stated "GCRA is considered a standard of care for selected patients with colorectal cancer". Depending on the answer to the above re: GCRA, it is the standard of care for all patients with CRC to have their cancer risk assessed by collection, analysis and comparison of their clinical information to confirm their eligibility for germline testing. NCCN guidelines indicate germline testing should be considered in all patients with CRC. If the primary intent of this work is to validate PREMM5 as a strategy to be applied in resource constrained geographies for selectively allotting germline testing only to those patients at predicted higher relative risk for having a P/LP, this should be clarified at the outset.

R= We appreciate this important clarification. We have revised the statement to reflect current guidelines, acknowledging that germline testing should be considered in all patients with colorectal cancer. We further clarify that, in resource-constrained settings, tools such as PREMM5 may help prioritize patients for testing when universal access is not feasible.

3. In the Methods the inclusion criteria are outlined, e.g. >=18 yo, diagnosis of CRC, age at diagnosis <50yo, etc. These inclusion criteria represent a "pre-screen" of patients with CRC which has implications for the total number/percentage of patients with P/LP identified after subsequent application of the screening strategies applied. The fact that these inclusion criteria functioned to pre-screen the total population of patients with CRC at the two participating institutions and select only certain qualifying CRC patients for the study needs to be explained in the text. Please add this to the Methods.

R= We agree with the reviewer that the inclusion criteria effectively functioned as a pre-screening step. We have now clarified this in the Methods section, explicitly stating that only patients meeting predefined clinical criteria were invited to participate, which enriched the study population for hereditary cancer risk.

4. To the comment above, how many patients with CRC were "pre-screened" using the inclusion criteria in order to obtain the 208 patients that were enrolled? i.e. what was the total population of patients with CRC seen at these two institutions during the enrollment timeframe of the study?

R= During the study period, a total of 1,081 patients with colorectal cancer were evaluated at the participating institutions, of whom 208 met the inclusion criteria and completed all study procedures. This information has now been added to the Methods section. This process is also illustrated in the newly added Figure 1 as a flowchart.

5. Methods paragraph 3 is noted patients were consented to the study then underwent GCRA. Was prescreening using inclusion criteria performed before or after obtaining consent, or were only those patients who met the inclusion criteria invited to give their consent to be part of the study? Here again, there is a need to define what it means that patients "underwent GCRA". Please clarify.

R= We have clarified that pre-screening based on clinical criteria was performed prior to obtaining informed consent, and only eligible patients were invited to participate.

6. Methods section paragraph 4 notes lack of genetic testing coverage for PMS2 exons 11-15 and PTEN promoter region. This should be reiterated in the Discussion as a limitation as it impacts interpretation of the overall P/LP prevalence in the tested population with some studies suggesting >10% of PMS2 P/LP occurring in exons 11-5.

R= We agree and have now included this in the Limitations section, acknowledging that incomplete coverage of PMS2 exons 11–15 and the PTEN promoter region may have led to underdetection of pathogenic variants; this statement is now supported by two additional references.

7. Also in Methods paragraph 4, second to last line indicates "...MSH2 and MSH6 were also analyzed for number variants...", where I believe "copy-number variants" is intended, please correct if so.

R= We thank the reviewer for noting this. The term has been corrected to “copynumber variants.”

8. Discussion section, first line states "individuals with CRC who met clinical criteria for GCRA". Again, what does GCRA mean? and what are the clinical criteria that were met for a patient to undergo GCRA? This needs to be clarified as it is currently unclear if the clinical criteria are the inclusion criteria, the criteria represented by each of the screening strategies (PREMM5, Amsterdam, Bethesda, IHC), NCCN criteria or something else.

R= We have revised the Discussion to ensure consistent and clear use of the term GCRA, explicitly referring to the predefined inclusion criteria and clinical risk assessment process used in this study.

9. Discussion section, second paragraph, "...77.6% of hereditary CRC cases were attributed to Lynch syndrome..." should be changed to, "77.6% of selected hereditary CRC cases...", as all patients were prescreened prior to enrollment and all enrolled patients were screened via PREMM5, Amsterdam, Bethesda or IHC prior to germline testing.

R= We agree and have revised the text to read “selected hereditary CRC cases” to reflect the pre-screened nature of the cohort.

Attachment Submitted filename: Response to Reviewers May 5.docx

# Peer review: Decision Letter 1

**DOI:** 10.1371/journal.pone.0354795.r003

**Authors:** Miquel Vall-llosera Camps

14 Jul 2026

Performance of PREMM5, Clinical Criteria, and Immunohistochemistry for MMR Proteins in Genetic Risk Assessment of Mexican patients with Colorectal Cancer

PONE-D-25-45234R1

Dear Dr. Chávarri-Guerra,

We’re pleased to inform you that your manuscript has been judged scientifically suitable for publication and will be formally accepted for publication once it meets all outstanding technical requirements.

Within one week, you’ll receive an e-mail detailing the required amendments. When these have been addressed, you’ll receive a formal acceptance letter and your manuscript will be scheduled for publication.

An invoice will be generated when your article is formally accepted. Please note, if your institution has a publishing partnership with PLOS and your article meets the relevant criteria, all or part of your publication costs will be covered. Please make sure your user information is up-to-date by logging into Editorial Manager at Editorial Manager® and clicking the ‘Update My Information' link at the top of the page. For questions related to billing, please contact billing support.

If your institution or institutions have a press office, please notify them about your upcoming paper to help maximize its impact. If they’ll be preparing press materials, please inform our press team as soon as possible -- no later than 48 hours after receiving the formal acceptance. Your manuscript will remain under strict press embargo until 2 pm Eastern Time on the date of publication. For more information, please contact onepress@plos.org.

Kind regards,

Miquel Vall-llosera Camps

Senior Staff Editor

PLOS One

Reviewers' comments:

Reviewer's Responses to Questions

**Comments to the Author**

1. If the authors have adequately addressed your comments raised in a previous round of review and you feel that this manuscript is now acceptable for publication, you may indicate that here to bypass the “Comments to the Author” section, enter your conflict of interest statement in the “Confidential to Editor” section, and submit your "Accept" recommendation.

Reviewer #2: All comments have been addressed

**********

2. Is the manuscript technically sound, and do the data support the conclusions?

The manuscript must describe a technically sound piece of scientific research with data that supports the conclusions. Experiments must have been conducted rigorously, with appropriate controls, replication, and sample sizes. The conclusions must be drawn appropriately based on the data presented.

Reviewer #2: Yes

**********

3. Has the statistical analysis been performed appropriately and rigorously?

Reviewer #2: Yes

**********

4. Have the authors made all data underlying the findings in their manuscript fully available?

The PLOS Data policy requires authors to make all data underlying the findings described in their manuscript fully available without restriction, with rare exception (please refer to the Data Availability Statement in the manuscript PDF file). The data should be provided as part of the manuscript or its supporting information, or deposited to a public repository. For example, in addition to summary statistics, the data points behind means, medians and variance measures should be available. If there are restrictions on publicly sharing data—e.g. participant privacy or use of data from a third party—those must be specified.

Reviewer #2: Yes

**********

5. Is the manuscript presented in an intelligible fashion and written in standard English?

PLOS ONE does not copyedit accepted manuscripts, so the language in submitted articles must be clear, correct, and unambiguous. Any typographical or grammatical errors should be corrected at revision, so please note any specific errors here.

Reviewer #2: Yes

**********

6. Review Comments to the Author

Please use the space provided to explain your answers to the questions above. You may also include additional comments for the author, including concerns about dual publication, research ethics, or publication ethics. (Please upload your review as an attachment if it exceeds 20,000 characters)

Reviewer #2: The author have adequately addressed my previously provided comments and recommendations, nothing further to add.

**********

7. PLOS authors have the option to publish the peer review history of their article (what does this mean?). If published, this will include your full peer review and any attached files.

If you choose “no”, your identity will remain anonymous but your review may still be made public.

**Do you want your identity to be public for this peer review?**  For information about this choice, including consent withdrawal, please see our Privacy Policy.

Reviewer #2: **Yes:** Edward D. Esplin

**********

# Editor report: Acceptance letter

**DOI:** 10.1371/journal.pone.0354795.r004

**Authors:** Miquel Vall-llosera Camps

PONE-D-25-45234R1

PLOS One

Dear Dr. Chávarri-Guerra,

I'm pleased to inform you that your manuscript has been deemed suitable for publication in PLOS One. Congratulations! Your manuscript is now being handed over to our production team.

At this stage, our production department will prepare your paper for publication. This includes ensuring the following:

* All references, tables, and figures are properly cited

* All relevant supporting information is included in the manuscript submission,

* There are no issues that prevent the paper from being properly typeset

You will receive further instructions from the production team, including instructions on how to review your proof when it is ready. Please keep in mind that we are working through a large volume of accepted articles, so please give us a few days to review your paper and let you know the next and final steps.

Lastly, if your institution or institutions have a press office, please let them know about your upcoming paper now to help maximize its impact. If they'll be preparing press materials, please inform our press team within the next 48 hours. Your manuscript will remain under strict press embargo until 2 pm Eastern Time on the date of publication. For more information, please contact onepress@plos.org.

You will receive an invoice from PLOS for your publication fee after your manuscript has reached the completed accept phase. If you receive an email requesting payment before acceptance or for any other service, this may be a phishing scheme. Learn how to identify phishing emails and protect your accounts at https://explore.plos.org/phishing.

If we can help with anything else, please email us at customercare@plos.org.

Thank you for submitting your work to PLOS ONE and supporting open access.

Kind regards,

PLOS ONE Editorial Office Staff

on behalf of

Dr. Miquel Vall-llosera Camps

Staff Editor

PLOS One
